# Curcumin-primed periodontal ligament stem cells-derived extracellular vesicles improve osteogenic ability through the Wnt/β-catenin pathway

**DOI:** 10.3389/fcell.2023.1225449

**Published:** 2023-09-28

**Authors:** Qian Lan, Jiadong Cao, Xueting Bi, Xin Xiao, Dongsheng Li, Yilong Ai

**Affiliations:** ^1^ Foshan Stomatology Hospital, School of Medicine, Foshan University, Foshan, Guangdong, China; ^2^ Deja Laboratory, Foshan, Guangdong, China

**Keywords:** curcumin, extracellular vesicles, periodontal ligament stem cells, oteogenesis, Wnt/β-catenin

## Abstract

**Introduction:** Curcumin has broad application prospects in the prevention and treatment of periodontal diseases. Periodontal ligament stem cell-derived extracellular vesicles (PDLSC-EV) can effectively promote periodontal tissue regeneration and possess good drug delivery capability. Superior pharmacological effects can be exerted using PDLSC-EV as a curcumin carrier.

**Methods:** In the present study, we constructed curcumin-primed PDLSCs-derived extracellular vesicles (Cur-PDLSC-EV) from cell culture supernatants of curcumin-pretreated PDLSCs by ultracentrifugation and investigated their effects on the proliferation, migration, and osteogenic ability of PDLSCs and the corresponding downstream molecular pathways.

**Results:** Both Cur-PDLSC-EV and PDLSC-EV promoted osteoblast proliferation and migration. Compared with PDLSC-EV, Cur-PDLSC-EV possessed a more potent pro-osteogenic ability. Moreover, the improved osteogenesis of Cur-PDLSC-EV was related to the activation of the Wnt/β-catenin signaling pathway.

**Conclusion:** This study suggests that Cur-PDLSC-EV can promote osteogenic differentiation by activating Wnt/β-catenin, providing reference bases for the treatment of periodontal diseases.

## 1 Introduction

Periodontitis is a chronic infectious disease with periodontal support tissue damage and loss caused by multiple factors, and it is the primary reason leading to adult teeth loss. With the progression of the disease, alveolar bone resorption and teeth loss or falling out occurs, severely affecting the quality of life of people (Slots, 2017; Yang et al., 2021). Once the alveolar bone tissues undergo destruction and resorption, it is very difficult for them to self-rebuild and regenerate. Currently, implant therapies with bone substitute materials are mainly employed clinically for alveolar bone defects, whereas there are still drawbacks, such as limited bone amount, low biocompatibility, susceptibility to infection, and induction of inflammatory response (Li et al., 2021; Sculean et al., 2015).

Compared with the traditional methods of treatment, regenerative tissue engineering based on stem cells proposes new ideas for periodontal tissue reconstruction and regeneration (Amato et al., 2022; Hu et al., 2018). Particularly, the periodontal ligament stem cells (PDLSCs) existing in periodontal ligaments have become important seed cells in the processes of alveolar bone regeneration and restoration (Calabrese, 2021; Tomokiyo et al., 2019). Research in recent years has indicated that the enormous potentials of PDLSCs in regenerative medicine are achieved to a great extent depending on their secreted active constituents such as extracellular vesicles and cytokines (Iwasaki et al., 2021; Rajan et al., 2016). Periodontal ligament stem cell-derived extracellular vesicles (PDLSC-EV) are a type of natural lipid nanosized small vesicles that are secreted by PDLSCs, which incorporate active components, such as functional protein, mRNA, and microRNA, and can be delivered to the recipient cells (Hua et al., 2021; Zhao et al., 2022). PDLSC-EV have crucial biological functions, such as promoting cellular proliferation, reducing apoptosis, facilitating angiogenesis, and improving tissue repair potential, and possess good application prospects in the aspects of regulating tissue regeneration (Chiricosta et al., 2020; Kang et al., 2018; Liu et al., 2020). Previous research from our laboratory revealed that PDLSC-EV promoted the proliferation and migration capacity of osteoblasts and enhanced COL1, Runx2, and ALP mRNA expression and mineralization *in vitro* (Lan et al., 2022).

Moreover, as natural nanoscale vesicles, PDLSC-EV exhibit characteristics, such as being less immunogenic, well targeting, and highly safe, which render them suitable promising pharmaceutical carriers that can be used for the delivery of nucleic acids, proteins, and small-molecule drugs (Liu et al., 2022; Sun et al., 2021; Yeo et al., 2013). The small-molecule compound curcumin is derived from the rhizome of *Curcuma longa*, a plant belonging to the *Ziangiberaceae* family. Curcumin has various biological activities, including antibacterial, anti-inflammatory, antioxidant, and immunomodulatory activities (Kotha and Luthria, 2019; Nelson et al., 2017). However, due to its poor water solubility, unstable structure, and low bioavailability, its applications in the treatment of periodontal disease are largely restricted (Forouzanfar et al., 2020; Lei et al., 2022). Accumulating evidence suggests that extracellular vesicles, as the drug carrier of curcumin, can increase the drug stability and improve the drug efficacy of curcumin *in vivo* without obvious adverse reactions.

In the present study, we cultivated PDLSCs on large-scale by adding curcumin to PDLSCs culture systems, derived the curcumin-primed periodontal ligament stem cell extracellular vesicles (Cur-PDLSC-EV) through ultracentrifugation, and investigate their effects on the proliferation, migration, and osteogenic ability of PDLSCs and the associated possible mechanisms. We provided evidence that the pro-osteogenic differentiation action of Cur-PDLSC-EV is potentially mediated via activating Wnt/β-catenin signaling pathway.

## 2 Materials and methods

### 2.1 Cell culture

PDLSCs were cultured using methods described in a previous publication (Lan et al., 2022). In brief, the cryopreserved PDLSCs were resuscitated and subsequently resuspended in a mesenchymal stem cell complete medium (Cyagen, China). After inoculation, the cells were cultured in an incubator at 37°C. When the cultured cells reached a confluency of 80%, they were passaged at a ratio of 1:2.

### 2.2 Isolation, purification, and characterization of Cur-PDLSC-EV and PDLSC-EV

For the preparation of Cur-PDLSC-EV, the PDLSCs were cultured in an exosome-free medium containing 10 μg/mL curcumin for 24 h. The supernatants were collected and centrifuged at 300 g for 5 min at 4°C to remove the dead cells. The collected supernatants were then centrifuged at 10,000 g for 30 min at 4°C to remove cellular debris. The supernatants were transferred to a 38.5 mL ultracentrifugation tube (Beckman Coulter, United States), and centrifuged at 100,000 g for 70 min. Subsequently, the pellets were resuspended and added to a 5.5 mL ultracentrifugation tube (Beckman Coulter, United States). At the bottom of the ultracentrifugation tube, 500 µL Exojuice (WeinaBio, China) was added, followed by adding PBS to fill up. After centrifuging at 100,000 g for 70 min, 200 µL liquid was removed at the bottom, and then 200 µL liquid was pipetted, which contained extracellular vesicles of high purity, and stored at −80°C before use.

PDLSC-EV were isolated using methods previously described in the literature (Lan et al., 2022). Briefly, after culturing the PDLSCs in the exosome-free medium for 24 h, the cell supernatants were collected. Extracellular vesicles were isolated using the ultracentrifugation method, and purified using the Exojuice–Exosome Purification and Concentration Kit (WeinaBio, China).

The particle size and electric potential of extracellular vesicles were analyzed using a laser particle size analyzer. The morphology of extracellular vesicles was observed using a transmission electron microscope. Western blotting was used to detect the exosome-related specific proteins (CD9, CD63, CD81, and TSG101).

### 2.3 Internalization of extracellular vesicles

To confirm the absorption of Cur-PDLSC-EV and PDLSC-EV by cells, the extracellular vesicles were labeled with a red-fluorescent dye (DiI, Solarbio, China) as described previously in the literature (Lan et al., 2022). Briefly, EV were treated with 5 µl of 10 µM DiI-labeling dye in 1 ml serum-free medium and incubated for 30 min at 37°C. EV suspension was ultracentrifuged at 100,000 g for 70 min at 4°C. They were then incubated with the PDLSCs at 37°C for 3 h, and fixed in 4% paraformaldehyde for 15 min. FITC- phalloidin (Yeasen, China) was used to stain the cytoskeleton for 45 min, and DAPI (Solarbio, China) staining was used for the nuclei. The red signals in the cells were detected using a fluorescence microscope (Mshot, China).

### 2.4 Cell counting kit-8 (CCK-8)

PDLSCs were inoculated in a 96-well plate at a density of 5,000 cells per well, with five parallel sets designed for each group. After growing for 24 h, the original culture medium was removed, and 50 μg/mL Cur-PDLSC-EV or PDLSC-EV were added. After an additional 48 h of culture, the culture medium was removed, and 100 µL fresh culture medium containing 10% CCK-8 reagent (APExBIO, United States) was added. After culturing in a 37°C incubator for 1.5 h, the absorbance at 450 nm was measured using a microplate reader (Thermo, United States), and the data were recorded.

### 2.5 Transwell

The cells were resuspended after digestion, and the suspension was diluted to 5 × 10^5^ cells/mL. Approximately 100 µL of the suspension was added to the upper chambers of the Transwell (Corning, United States), and 50 μg/mL PDLSC-EV or Cur-PDLSC-EV was added to the lower chambers. After 24 h of culture, the culture media in the upper and lower compartments were removed. After washing with PBS three times, 1 mL of 4% paraformaldehyde was added to the upper and lower compartments to fix for 30 min, washed again with PBS three times, and then 1 mL crystal violet was added for 30 min to stain. The excess dyes were subsequently removed, followed by washing with PBS three times. Subsequently, the cells in the upper chambers were gently wiped with a cotton swab, and the inserts were then properly air-dried. The migration results were observed and recorded under a microscope.

### 2.6 Alizarin red S (ARS) and alkaline phosphatase (AKP) staining

After digestion, the PDLSCs were seeded at a density of 2 × 10^4^ cells/well into a 24-well plate, and osteogenic differentiation medium (ODM, Cyagen, China) was added to induce osteogenesis. After 24 h, 50 μg/mL PDLSC-EV, Cur-PDLSC-EV or Cur-PDLSC-EV+(ICG-001 (10 μM) was added. ARS and AKP staining were performed after 7–10 days of culture-induced differentiation. Before staining, 4% paraformaldehyde (Beyotime, China) was added for fixing at room temperature for 30 min, followed by washing with PBS three times at 5 min each time. Subsequently, the ARS staining solution (Beyotime, China) and AKP staining solution (Beyotime, China) were added and stained for 5 and 30 min, respectively. The results were observed, pictures were taken, and recorded under a microscope.

### 2.7 RT-qPCR

RNA was extracted by using Trizol reagent and chloroform, precipitated by isopropanol, rinsed in 75% ethanol, and then resuspended in nuclease-free water. After measuring the concentration of the derived RNA, cDNA was synthesized by HiScript^®^ III RT SuperMix for qPCR (+gDNA wiper) (Vazyme, China). Quantitative PCR was performed using ChamQ Universal SYBR qPCR Master Mix (Vazyme, China) to detect the mRNA levels of osteogenesis-related genes COL1/ALP/RUNX2. β-Actin was used for the normalization of mRNA expression. All gene sequences were obtained from GenBank, and the primers were designed (Primer Premier 5) and synthesized (Sangon Biotech, China). Primer sequences are shown in Table 1.

**TABLE 1:** List of specific primers.

Gene	Forward primer	Reverse primer
COL1	CTG​ACC​TTC​CTG​CGC​CTG​ATG​TCC	GTC​TGG​GGC​ACC​AAC​GTC​CAA​GGG
ALP	GGA​CAT​GCA​GTA​CGA​GCT​GA	GCA​GTG​AAG​GGC​TTC​TTG​TC
Runx2	CGC​ATT​CCT​CAT​CCC​AGT​AT	GAC​TGG​CGG​GGT​GTA​AGT​AA
CCND1	CAA​GGC​CTG​AAC​CTG​AGG​AG	GAT​CAC​TCT​GGA​GAG​GAA​GCG
β-actin	GGC​ATC​CAC​GAA​ACT​ACA​TTC​AAT​TCC	GTA​CCA​CCA​GAC​AGC​ACT​GTG​TTG

### 2.8 Western blotting

BCA working solution and serially diluted BSA standards were prepared according to the reagent kit operation manual. A volume of 10 µL of the BSA standards and samples to be tested were added to 100 µL of the BCA working solution, mixed well, and then reacted at 37°C for 30 min. The sample concentration was measured using an ultra-micro spectrophotometer. Subsequently, 10 µL protein loading buffer was added to an aliquot of 50 µL exosome extract for protein denaturing at 95°C for 10 min. A quantity of 50 μg of total protein was separated through 10% SDS-PAGE, whose electrophoresis conditions were 90 V for 30 min and 120 V for 60 min. The proteins were then transferred to a PVDF membrane, with a regulated voltage of 60 V and a transfer time of 2 h. The membrane was blocked using 5% nonfat milk for 1 h. GAPDH/GSK3β/β-catenin antibodies (1:1000 dilution) were added and incubated overnight at 4°C. The membrane was then washed with 1 × TBST four times at 5 min each time. The corresponding horseradish peroxidase (HRP)-labeled secondary antibodies (a dilution ratio of 1:5000) were added and incubated for 1 h. The membrane was washed with TBST four times at 5 min each time. The protein expression was detected using the ECL chemiluminescent reagents, and the signals were developed in a chemiluminescence imaging system.

## 3 Results

### 3.1 Characterization of extracellular vesicles

**FIGURE 1:**
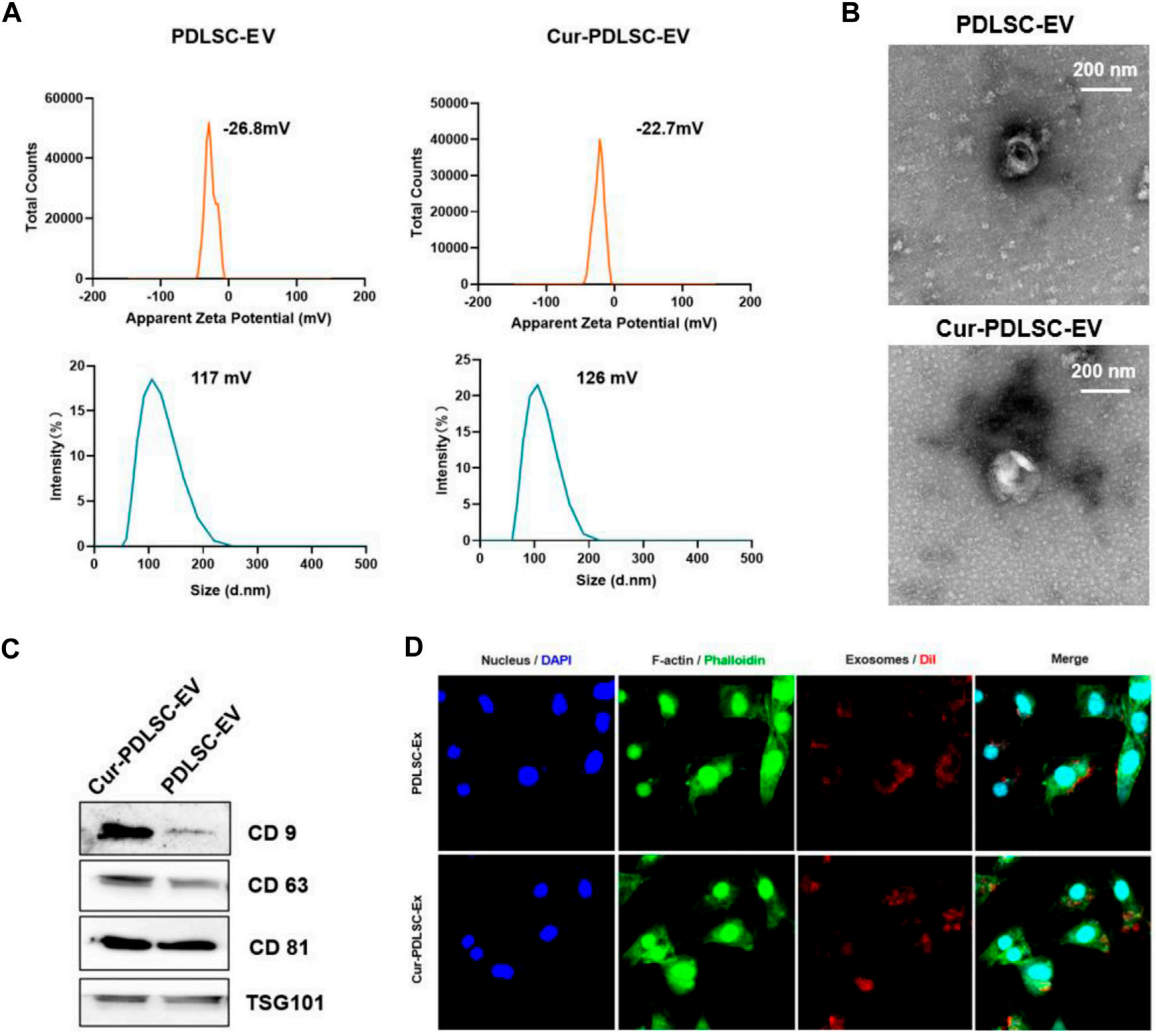
Characterization of Cur-PDLSC-EV and PDLSC-EV. **(A)** Particle size distribution and electric potential of Cur-PDLSC-EV and PDLSC-EV were measured by NTA. **(B)** Morphology of Cur-PDLSC-EV and PDLSC-EV under the transmission electron microscope. Scale bar, 100 nm. **(C)** Marker proteins (CD9, CD63, CD81 and TSG101) of extracellular vesicles detected using Western blotting. **(D)** Internalization of extracellular vesicles by PDLSCs. The red fluorescence indicates the DiI-labeled exosomes; the green fluorescence indicates the cytoskeleton; the blue fluorescence indicates the nucleus.

According to the laser particle size analyzer, PDLSC-EV had an average diameter of 117 nm and an average electric potential of −26.8 mV; Cur-PDLSC-EV exhibited an average diameter of 126 nm and an average electric potential of −22.7 mV (Figure 1A). The transmission electron microscopy revealed that both PDLSC-EV and Cur-PDLSC-EV displayed cup-shaped vesicles (Figure 1B). The Western blotting data showed that both PDLSC-EV and Cur-PDLSC-EV expressed CD81, CD63, CD9, and TSG101 proteins, and the CD9 and CD81 expression of Cur-PDLSC-EV were higher than those of PDLSC-EV (Figure 1C; Supplementary Material). After co-culture of DiI-labeled PDLSC-EV or Cur-PDLSC-EV with PDLSCs for 3 h, it could be observed under a fluorescence microscope that the extracellular vesicles had already been successfully internalized by PDLSCs (Figure 1D).

### 3.2 Cell proliferation and migration

**FIGURE 2:**
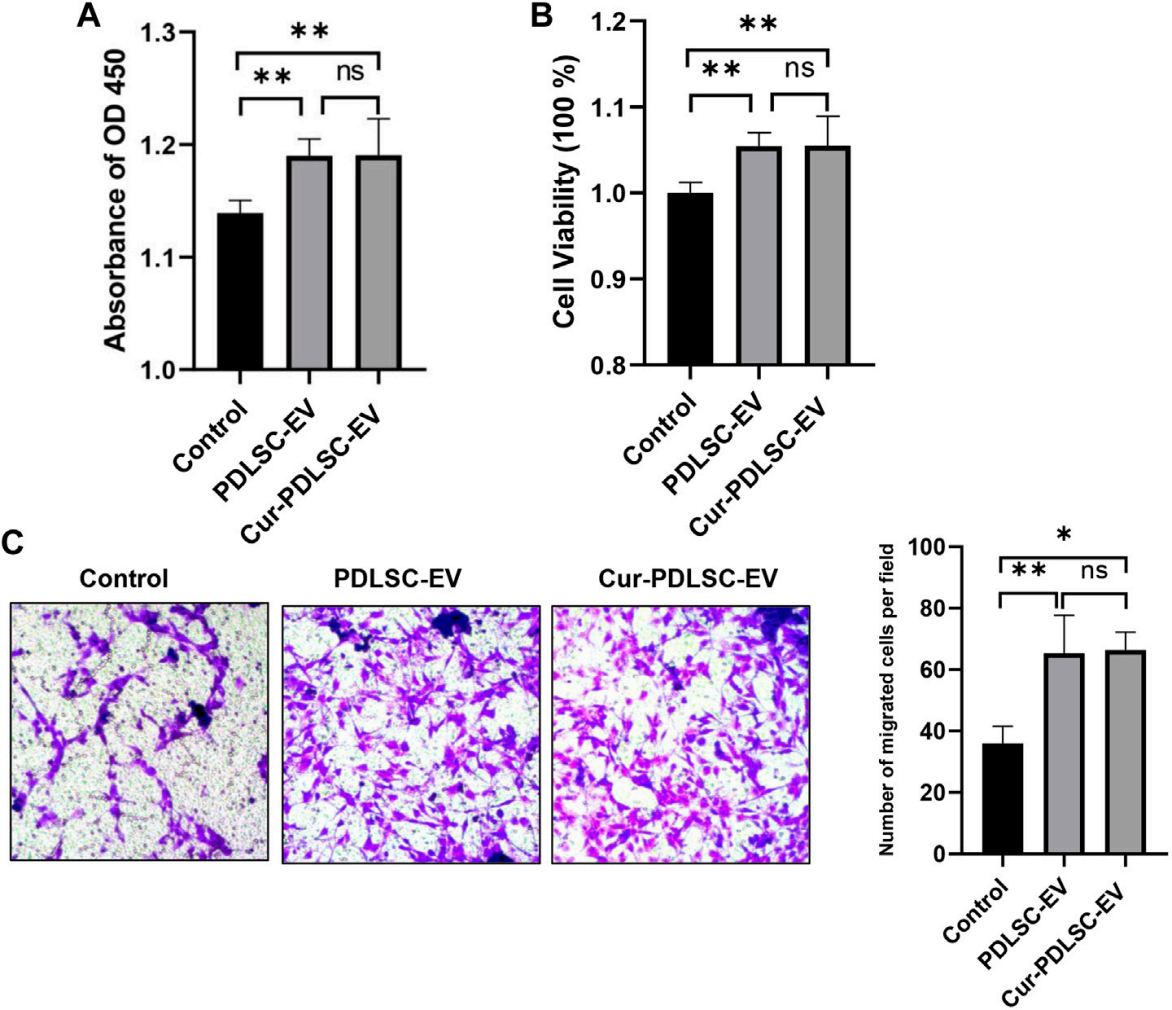
Effect of Cur-PDLSC-EV and PDLSC-EV on cell proliferation and migration. Using the CCK-8 test of OD_450_ **(A)** and cellular viability **(B)**, Cur-PDLSC-EV and PDLSC-EV promoted cell proliferation, and there was no difference between the two. **(C)** Transwell assay showing PDLSC-EV and Cur-PDLSC-EV facilitated cell migration, and there was no difference between the two.

The PDLSCs were treated with 50 μg/mL Cur-PDLSC-EV or PDLSC-EV, and after 48 h CCK-8 was added to measure the absorbance value at 450 nm. The OD_450_ and cellular viability results (Figures 2A, B) showed that PDLSC-EV and Cur-PDLSC-EV had promoting effects on the proliferation of PDLSCs, and they did not exhibit a difference in the enhanced activities between each other.

The transwell assay (Figure 2C) data showed that compared with the PBS control group, PDLSC-EV and Cur-PDLSC-EV possessed facilitative effects on the migration of PDLSCs, and there was no difference in the enhancement between the two.

### 3.3 ARS and AKP staining

**FIGURE 3:**
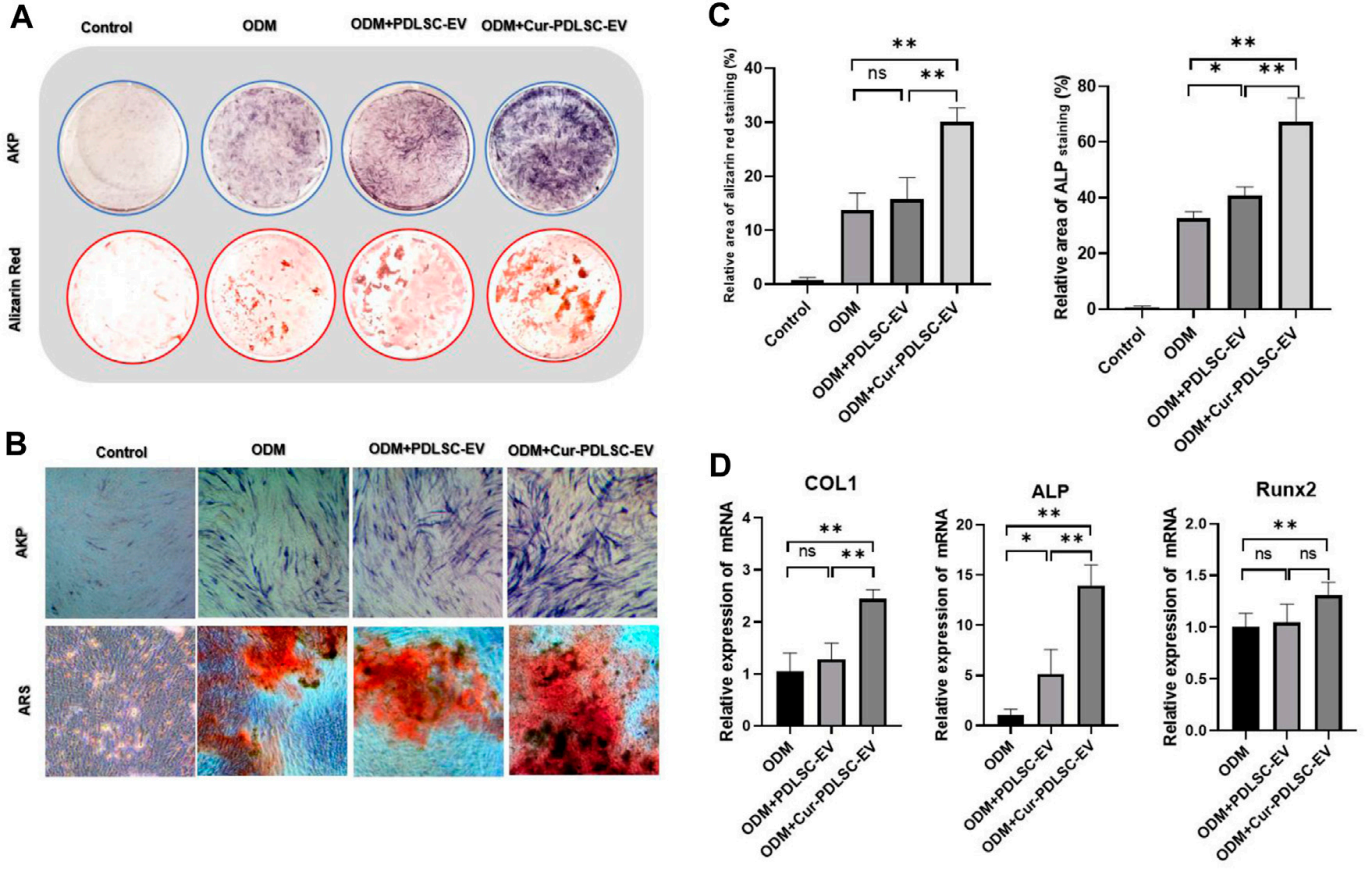
Effect of Cur-PDLSC-EV and PDLSC-EV on osteogenic differentiation. ARS and AKP staining in culture dishes **(A)** and under an inverted microscope **(B)**. **(C)** Result of ARS and AKP semi-quantitative analysis after osteogenic induction. **(D)** Relative mRNA levels of osteogenesis marker genes (COL1, Runx2, and ALP) detected using RT-qPCR.

**FIGURE 4:**
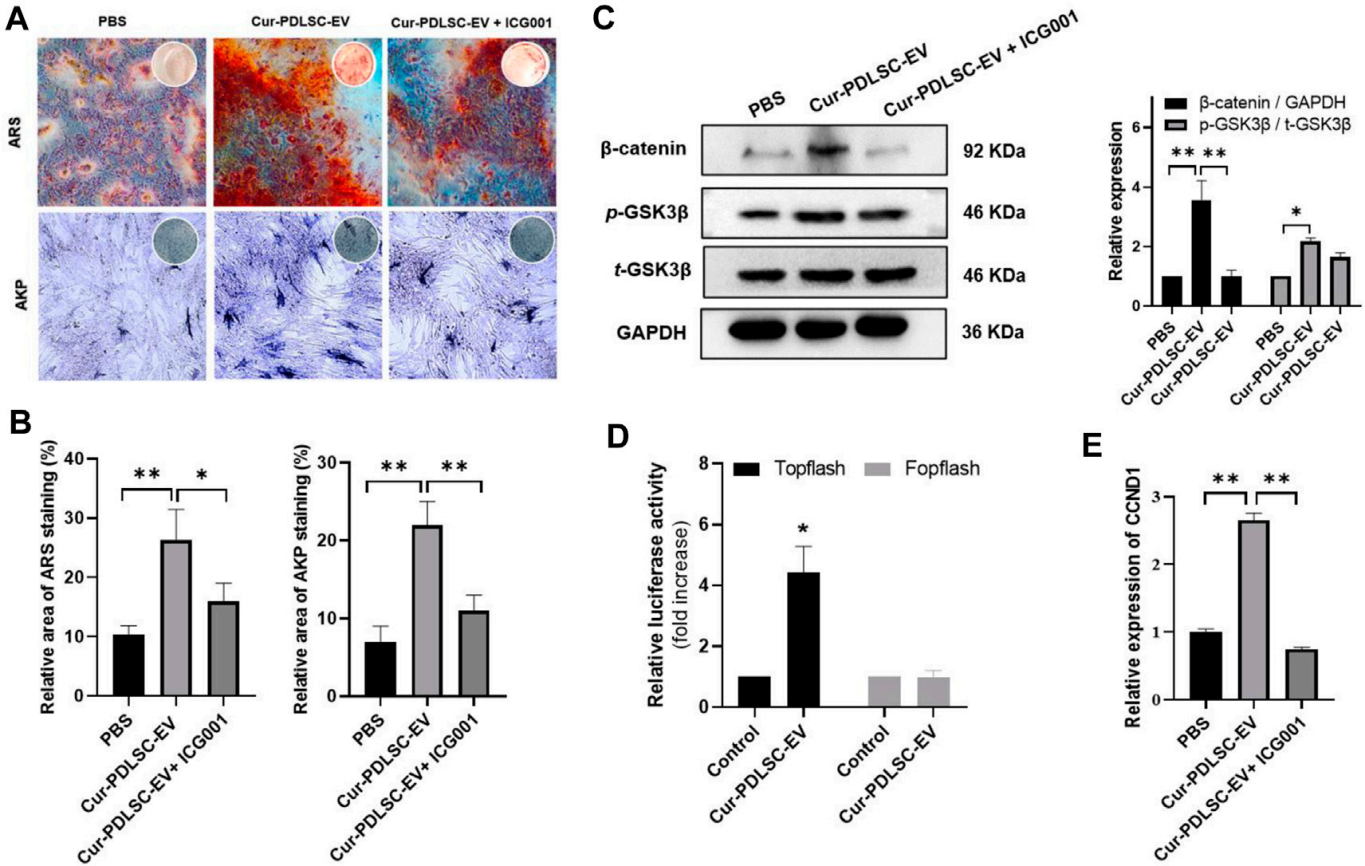
Effects of Cur-PDLSC-EV on Wnt/β-Catenin pathway. **(A)** The ability of Cur-PDLSC-EV and Wnt signaling pathway inhibitor ICG001 on osteogenic differentiation analyzed by Alizarin Red and AKP staining **(B)** Result of Alizarin Red and AKP semi-quantitative analysis after osteogenic induction. **(C)** Western blot assay for the expression levels of GSK3β and β-catenin in PDLSCs. **(D)** A luciferase assay based on a TOPflash/FOPflash reporter plasmid system was used to evaluate β-catenin-dependent transcriptional activity. **(E)** Relative mRNA levels of target gene of Wnt/β-Catenin pathway (CCND1) detected using RT-qPCR.

After osteogenic induction, the PDLSCs began to change from fibrous shape to polygonal, scale-like morphology. As the induction time increased, calcium nodules gradually formed, which after ARS staining could be visible as stained red mineral deposits. Based on the ARS and AKP staining results (Figures 3A–C), it was shown that either PDLSC-EV or Cur-PDLSC-EV promoted the osteogenic differentiation of PDLSCs, and Cur-PDLSC-EV had a better enhancement effect. Additionally, after adding ICG001, the pro-osteogenic ability of Cur-PDLSC-EV was reversed in PDLSCs (Figures 4A, B).

### 3.4 RT-qPCR

The RT-qPCR data revealed that PDLSC-EV had no effect on the expressions of osteogenesis-related genes COL1 and Runx2, while upregulating ALP gene expression (*p* < 0.05). Cur-PDLSC-EV had stimulating effects on the gene expression of all the osteogenesis-related genes COL1, Runx2, and ALP (*p* < 0.01). Comparing the PDLSC-EV and Cur-PDLSC-EV groups, there were extremely significant differences in COL1 and ALP gene expression (*p* < 0.01) (Figure 3D).

### 3.5 Effect of Cur-PDLSC-EV on the Wnt/β-catenin pathway

According to the Western blotting data, it was shown that Cur-PDLSC-EV upregulated the protein expression of *p-*GSK3β and β-catenin in PDLSCs. To further confirm that the activation of Wnt/β-catenin pathway is responsible for the progression of PDLSCs by Cur-PDLSC-EV treatment, PDLSCs were treated with Cur-PDLSC-EV in the presence of the inhibitor (ICG001) of Wnt/β-catenin pathway. Our results suggested that the Cur-PDLSC-EV-mediated activation of Wnt/β-catenin signaling could be blocked by ICG001, and the osteogenic differentiation was attenuated (Figure 4C; Supplementary Material). In addition, a luciferase assay based on a TOPflash/FOPflash reporter plasmid system was used to evaluate β-catenin-dependent transcriptional activity. The results demonstrated that Wnt/β-catenin signaling was activated following Cur-PDLSC-EV treatment (Figure 4D), accompanied by a significant increase in the mRNA levels of the downstream target gene CCND1 (Figure 4E). These results indicate that Cur-PDLSC-EV promoted osteogenesis by activating Wnt/β-catenin signaling pathway.

## 4 Discussion

In recent years, studies have discovered that stem cells derived from various tissues regulate cell proliferation, migration, and multilineage differentiation via the formation of secreting extracellular vesicles and by binding to recipient cells, thereby exerting regenerative and repairing capacity (Keshtkar et al., 2018; Yuan et al., 2022). In addition, stem cell extracellular vesicles as carriers loaded with nucleic acids, proteins, and small-molecule drugs enhance drug sustained release, stability, and bioavailability, which have exerted huge advantages in the investigations of tissue regeneration (Rezaie et al., 2022). Current research indicates that PDLSC-EV can act as the main information intermedia between PDLSCs and neighboring cells, deliver biologically active substances, and stimulate the regeneration and reconstruction of damaged tissues (Huang et al., 2022). As a “cell replacement” therapy with low immunogenicity, high safety, and easy storage and transportation, extracellular vesicles play pivotal roles in periodontium restoration and regeneration (Lei et al., 2022).

Curcumin is a natural diketone compound extracted from the rhizome of the *C. longa* plant, a member of the ginger family. It possesses multiple medicinal properties including antioxidant, anti-inflammatory, antibacterial, antifungal, antiviral, antiangiogenic, anticancer, and antiatherosclerotic (Tsuda, 2018). The advantageous attributes of curcumin in the treatments of diseases, including obesity, osteolysis, osteoporosis, and osteosarcoma, have been reported, and at the same time, it plays an important role in the aspects of stem cell osteogenic differentiation (Sanidad et al., 2019). Numerous studies have shown that curcumin helps increase bone density, strengthen bone microstructure, decrease oophorectomy-induced bone destruction, and repress osteoporosis and osteoarthritis (Cho et al., 2013; Daily et al., 2016; Riva et al., 2017; Yang et al., 2011).

However, the disadvantages of curcumin, including low water solubility, poor stability, and low bioavailability, considerably limit its widespread application in clinics. Using extracellular vesicles as delivery vehicles of curcumin could increase the solubility, stability, and bioavailability of curcumin, enhance the delivery of curcumin to activated monocytes, and elevate its anti-inflammatory activities (Farooqi et al., 2018; Kalani and Chaturvedi, 2017a; Oskouie et al., 2019). Vashisht et al. (2017) found that compared with free curcumin, curcumin encapsulated in milk extracellular vesicles had higher stability, protected curcumin against the impact of the digestive system environment, and was able to cross the intestinal barrier. Kalani and Chaturvedi (2017) demonstrated that curcumin-primed exosomes had neuroprotective effects because of their antilipidemic, antioxidative, and anti-inflammatory properties. Curcumin-primed exosomes could also reverse lipopolysaccharide-induced expression of pro-inflammatory genes in buffalo granulosa cells, alleviating the dysfunction of granulosa cells (Vashisht et al., 2018). In the present study, we observed that both Cur-PDLSC-EV and normal PDLSC-EV significantly increased cell proliferation and migration, and Cur-PDLSC-EV had a more potent pro-osteogenic ability.

**FIGURE 5:**
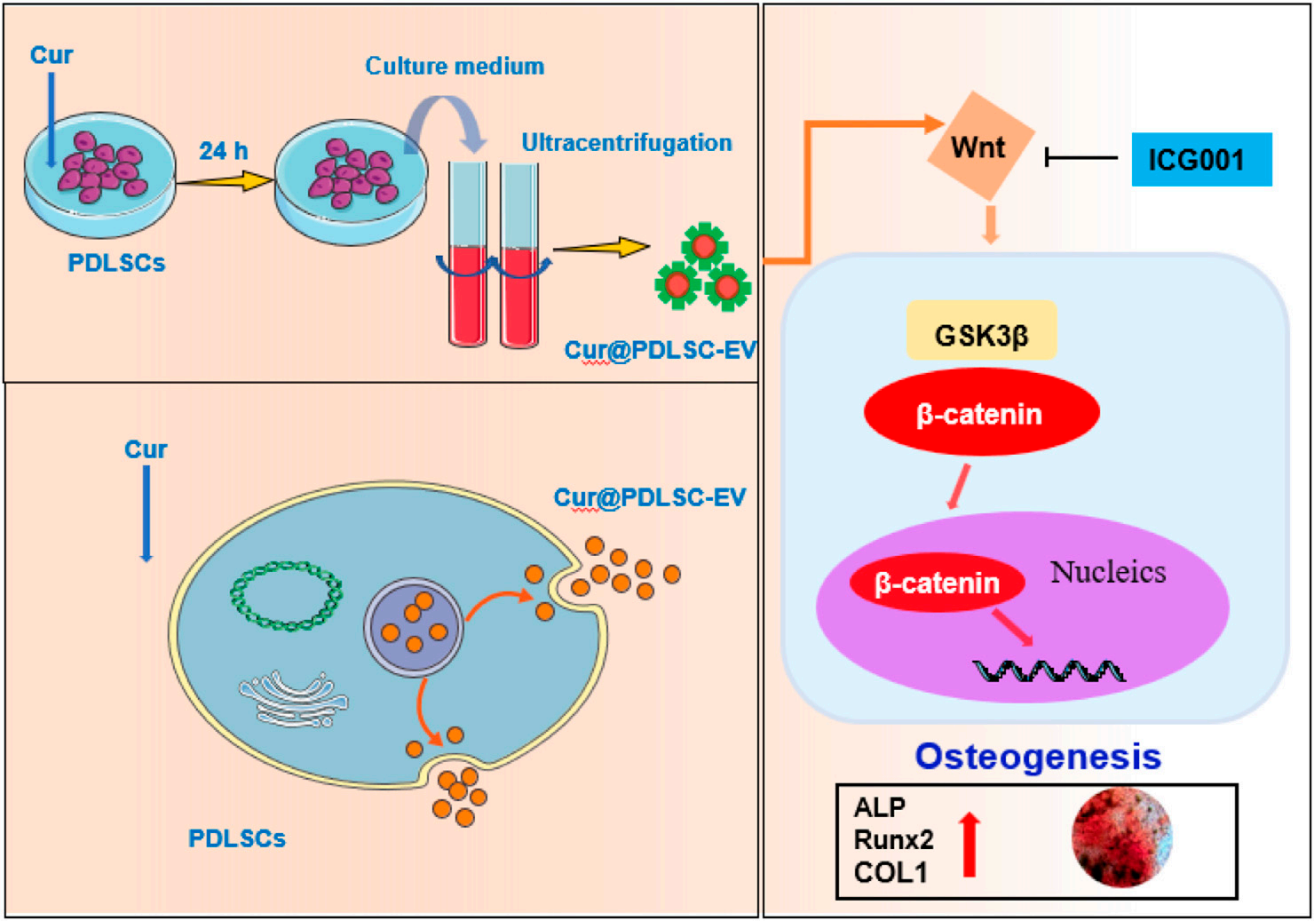
Schematic diagram of the mechanism of osteogenic differentiation induced by Cur-PDLSC-EV.

Previous studies demonstrated the Wnt/β-catenin pathway was one of the most important pathways in osteogenesis (Vashisht et al., 2018), we observed the effects of Cur-PDLSC-EV on Wnt/β-catenin signaling pathway, our results of Western blotting showed that Cur-PDLSC-EV significantly increase protein levels of p-GSK3β and β-catenin in PDLSCs. When we treated PDLSCs with ICG001, an inhibitor of Wnt/β-catenin pathway, we observed that the pro-osteogenic ability was reversed when Wnt/β-catenin signaling was inhibited, confirming its role in osteogenic differentiation of PDLSCs. Our study found that Cur-PDLSC-EV had a more potent pro-osteogenic ability, and the improved osteogenesis by Cur-PDLSC-EV was closely related to the Wnt/β-catenin signaling pathway (Figure 5).

## 5 Conclusion

Our study found that Cur-PDLSC-EV could promote the proliferation, migration, and osteogenic differentiation of PDLSCs. These functions were achieved by activating the Wnt/β-catenin signaling pathway. These results provide a reference basis for further research and clinical treatments of periodontal diseases and repair of the bone defect. However, it was not clear which contents were delivered by Cur-PDLSC-EV, specifically to exert their functions. As follow-ups, in-depth analyses will be further performed for the components of Cur-PDLSC-EV, and the key molecules in extracellular vesicles modulating the cellular functions of PDLSCs will be identified to provide clues for the further elucidation of the molecular mechanisms.

## Data Availability

The datasets presented in this study can be found in online repositories. The names of the repository/repositories and accession number(s) can be found below: https://we.tl/t-BaY6UePz5s.

## Abbreviations

PDLSCs, periodontal ligament stem cells; PDLSC-EV, periodontal ligament stem cell-derived extracellular vesicles; Cur-PDLSC-EV, curcumin-primed periodontal ligament stem cell-derived extracellular vesicles; CCK-8, cell counting kit-8; ARS, alizarin red S; AKP, alkaline phosphatase; ODM, osteogenic differentiation medium; HRP, horseradish peroxidase.
