# Supplementary material for: Curcumin-primed periodontal ligament stem cells-derived extracellular vesicles improve osteogenic ability through the Wnt/β-catenin pathway
Source: Front Cell Dev Biol. 2023 Sep 28;11:1225449. doi: 10.3389/fcell.2023.1225449 (PMC10568008; doi:10.3389/fcell.2023.1225449)
Supplement: Supplementary file 1 [file Image1.pdf]

Exosomal marker proteins detected using western blotting.

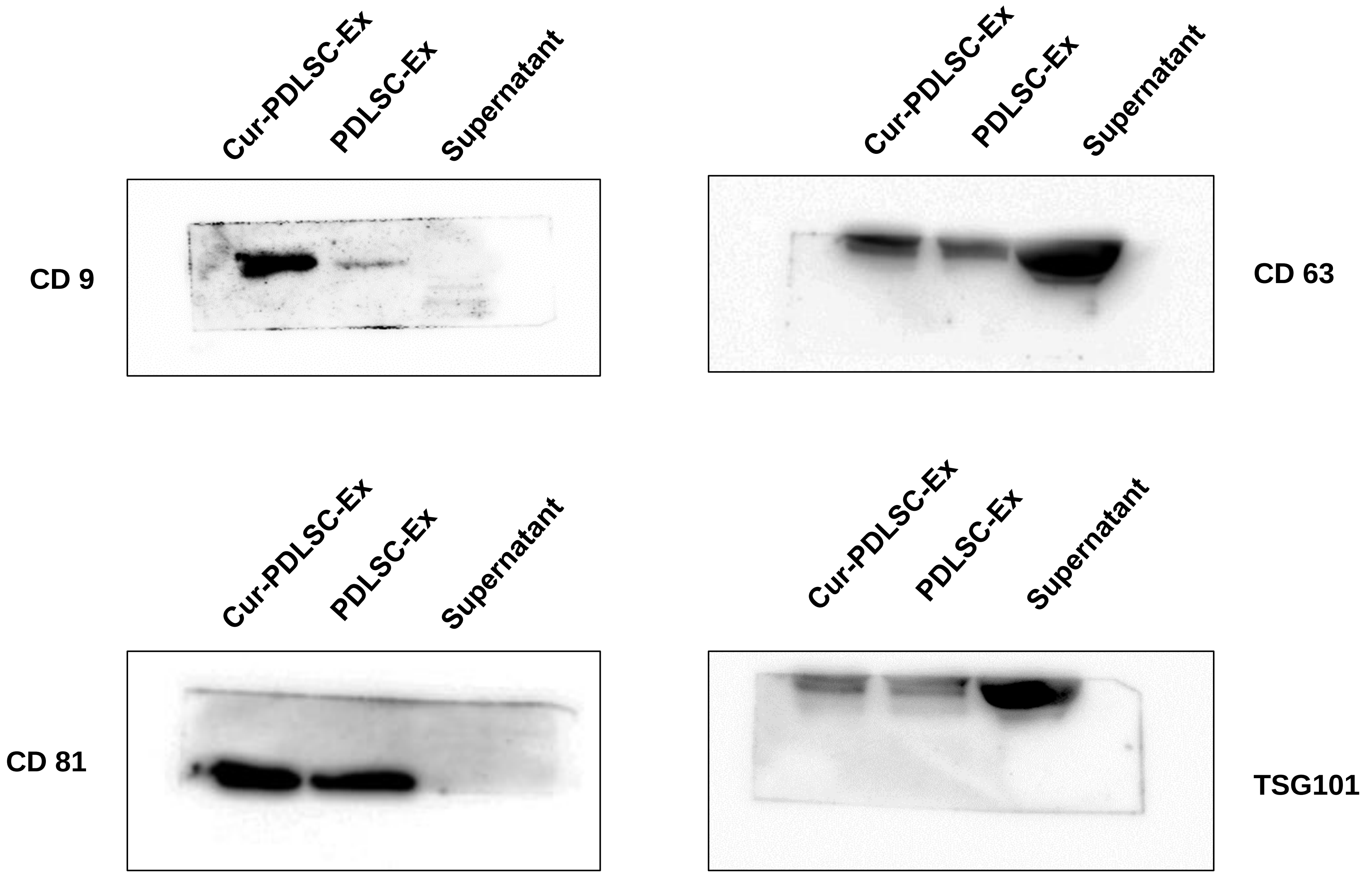

Western Blot Instructions and Original Images

Western blot assay for the expression levels of GSK3β and β-catenin in PDLSCs.

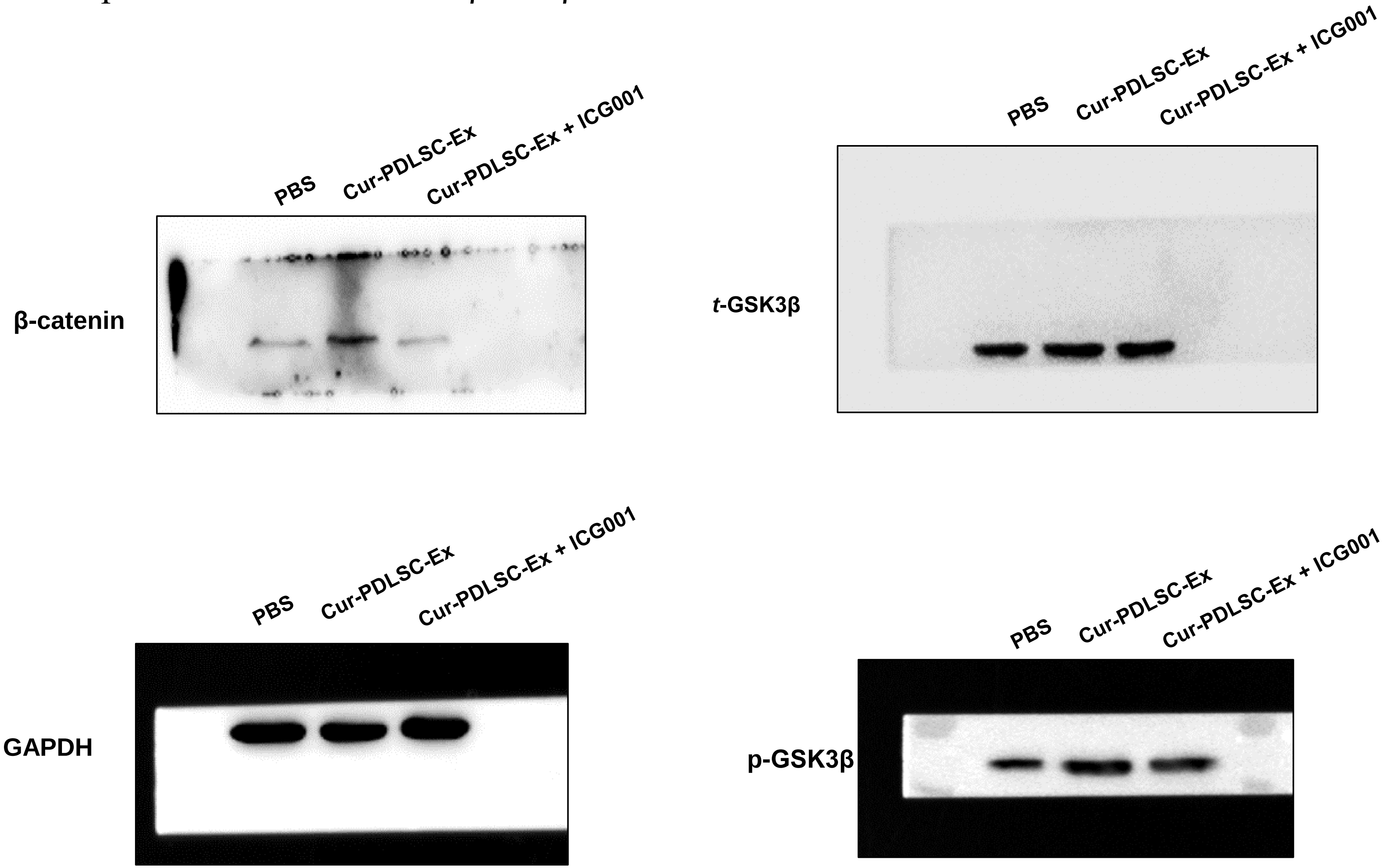

Western Blot Instructions and Original Images
